# Supplementary material for: The Construction and Exploration of a Comprehensive MicroRNA Centered Regulatory Network in Foxtail Millet (Setaria italica L.)
Source: Front Plant Sci. 2022 May 6;13:848474. doi: 10.3389/fpls.2022.848474 (PMC9121102; doi:10.3389/fpls.2022.848474)
Supplement: Supplementary file 4 [file Data_Sheet_4.zip › Sit-miR2275b_Seita.9G493100.1_2782_TPlot.pdf]

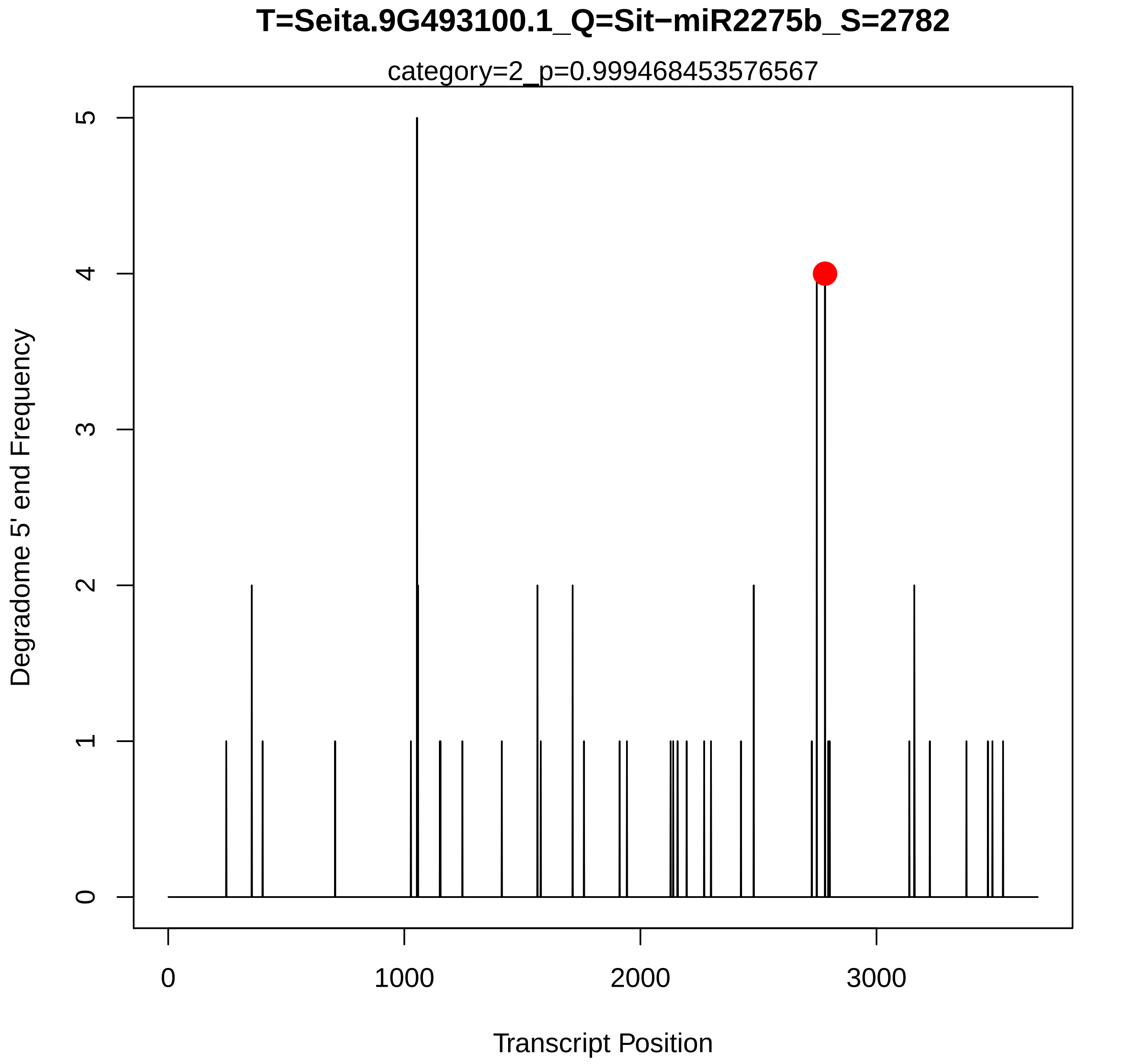

T=Seita.9G493100.1_Q=Sit−miR2275b_S=2782
category=2_p=0.999468453576567
Degradome 5' end Frequency
Transcript Position
0
1
2
3
4
5
0
1000
2000
3000
